# Supplementary material for: The impact of epilepsy surgery on the structural connectome and its relation to outcome
Source: Neuroimage Clin. 2018 Jan 31;18:202–14. doi: 10.1016/j.nicl.2018.01.028 (PMC5987798; doi:10.1016/j.nicl.2018.01.028)
Supplement: Supplementary file 1 — Supplementary material [file mmc1.pdf]

Supplementary Material

The impact of epilepsy surgery on the structural connectome and its relation to outcome

Peter N Taylor[1,2,3^], Nishant Sinha[1,2], Yujiang Wang[1,2,3], Sjoerd B Vos[4,5], Jane de Tisi[3], Anna Miserocchi[3], Andrew W McEvoy[3], Gavin P Winston[3,5*], John S Duncan[3,5*]

Supplementary Figure S1

Inferred percentage of streamlines removed post-surgery for both atlases is not significantly different between outcome groups (permutation test for difference between mean).

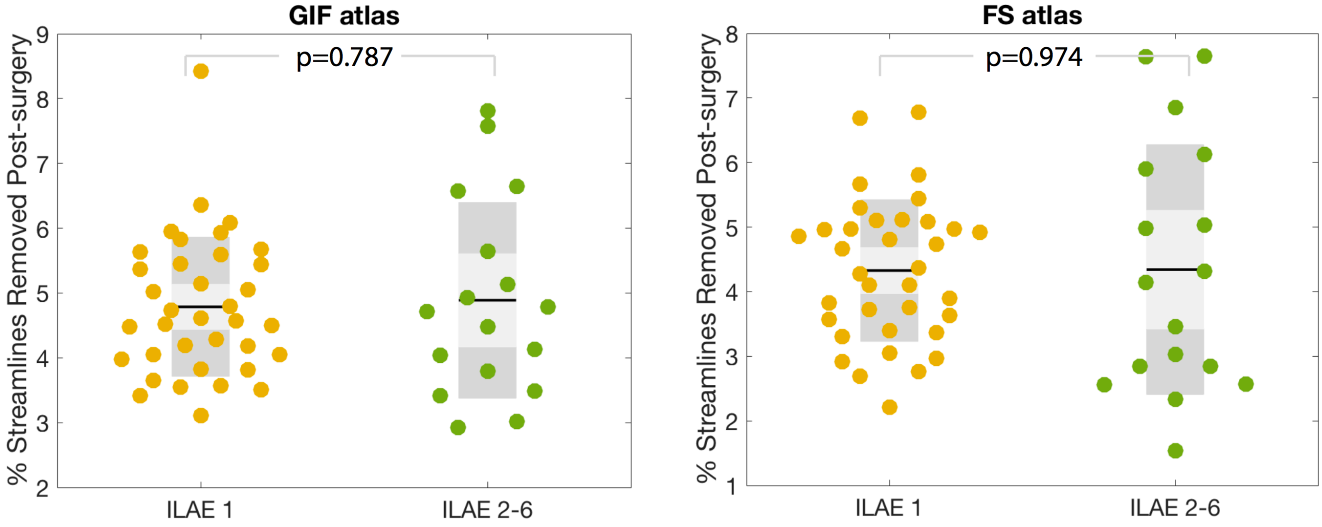

Supplementary Figure S2

Volume change & significance plots for the GIF derived atlas.

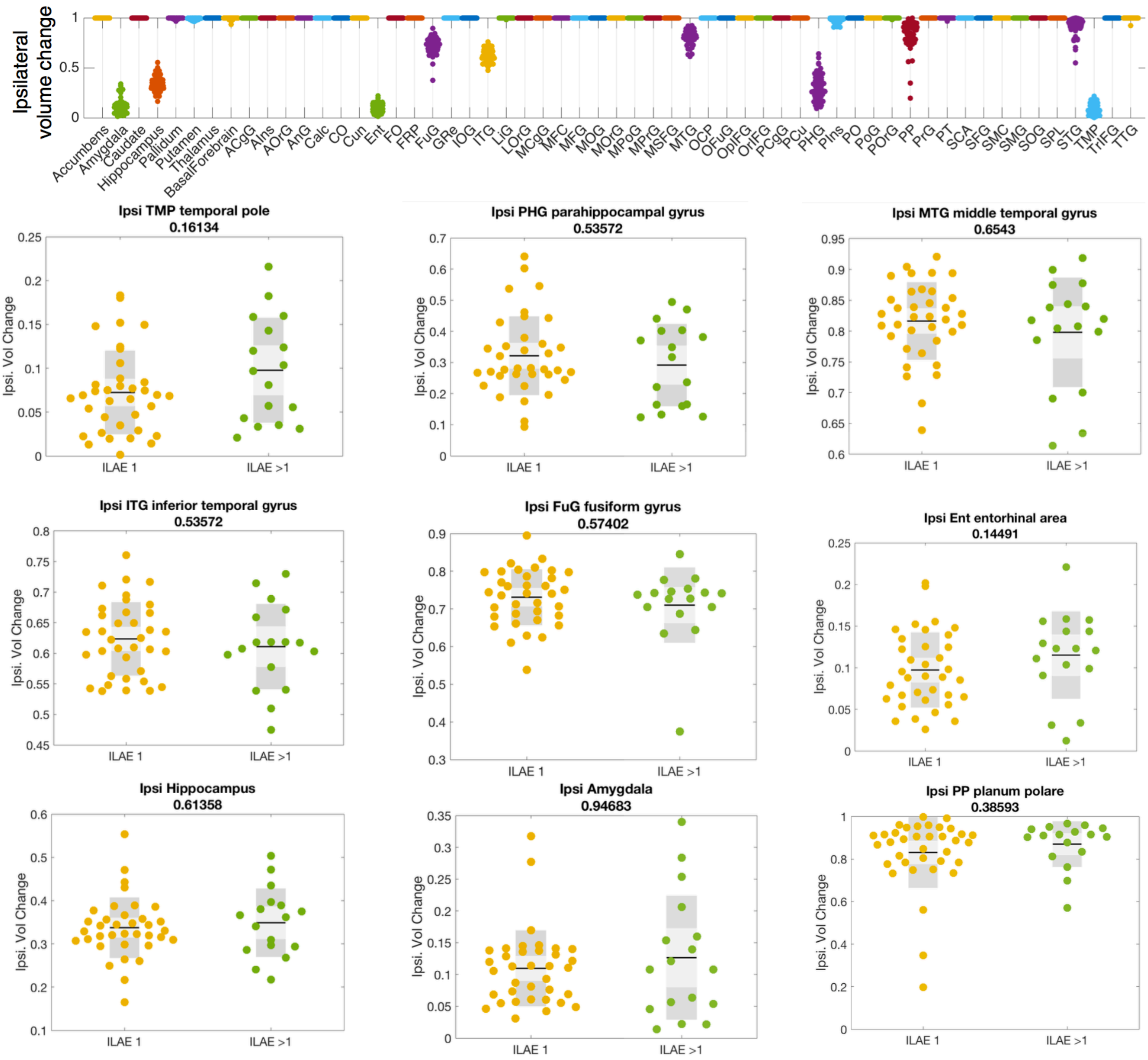

Supplementary Figure S3

Volume change & significance plots for the Freesurfer derived atlas.

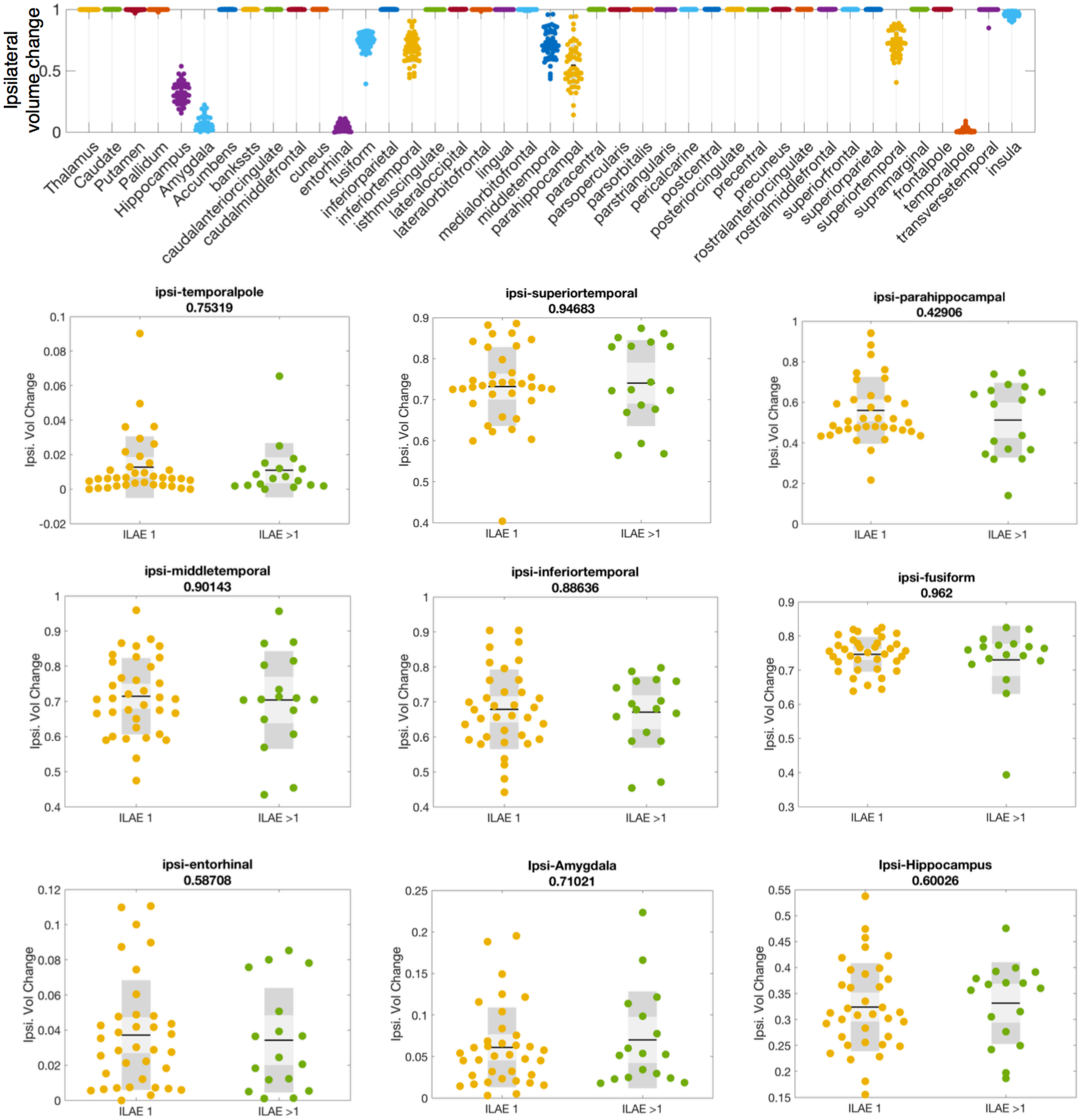

Supplementary Figure S4

Region strength for FreeSurfer derived atlas.

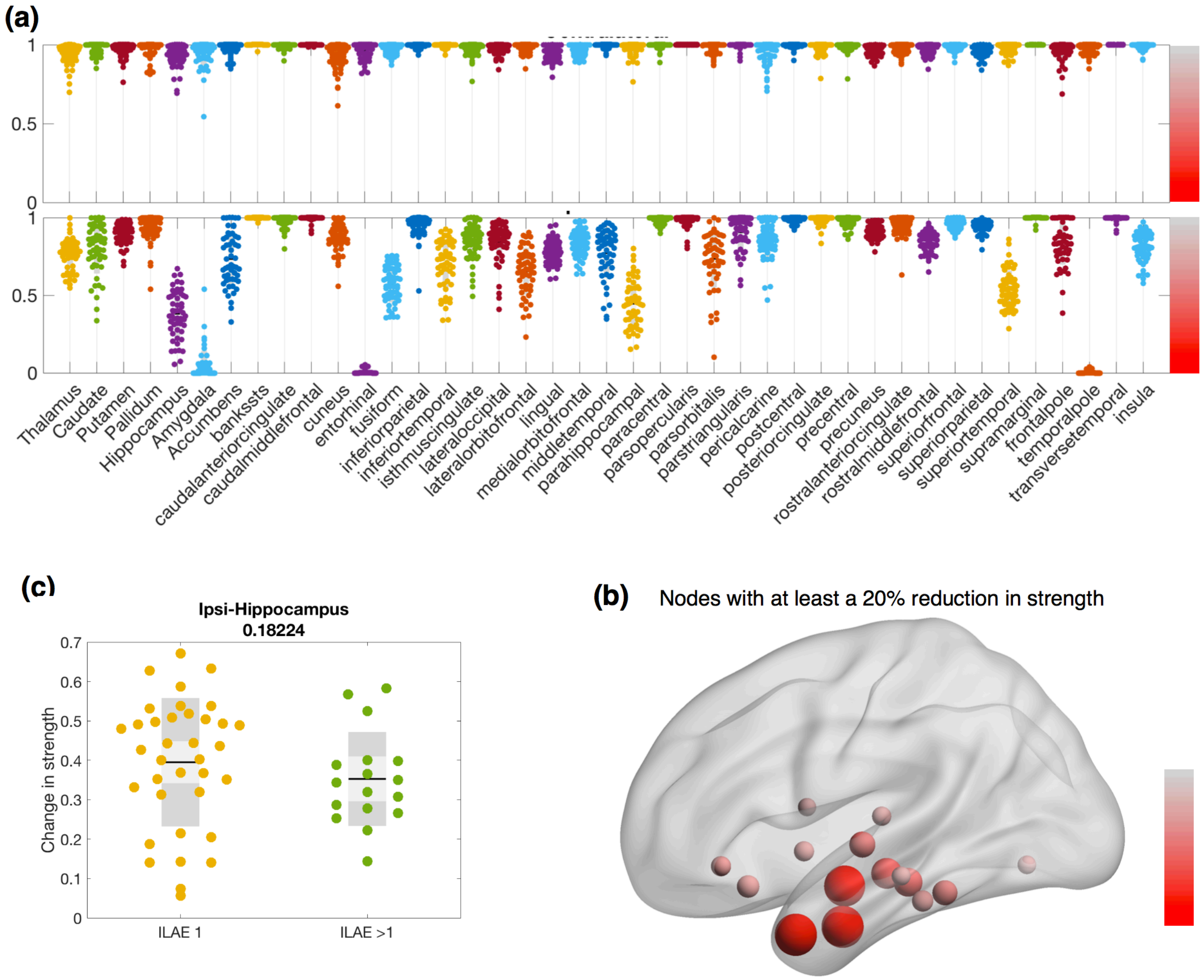

Supplementary Figure S5

Region betweenness centrality for using the FreeSurfer derived atlas.

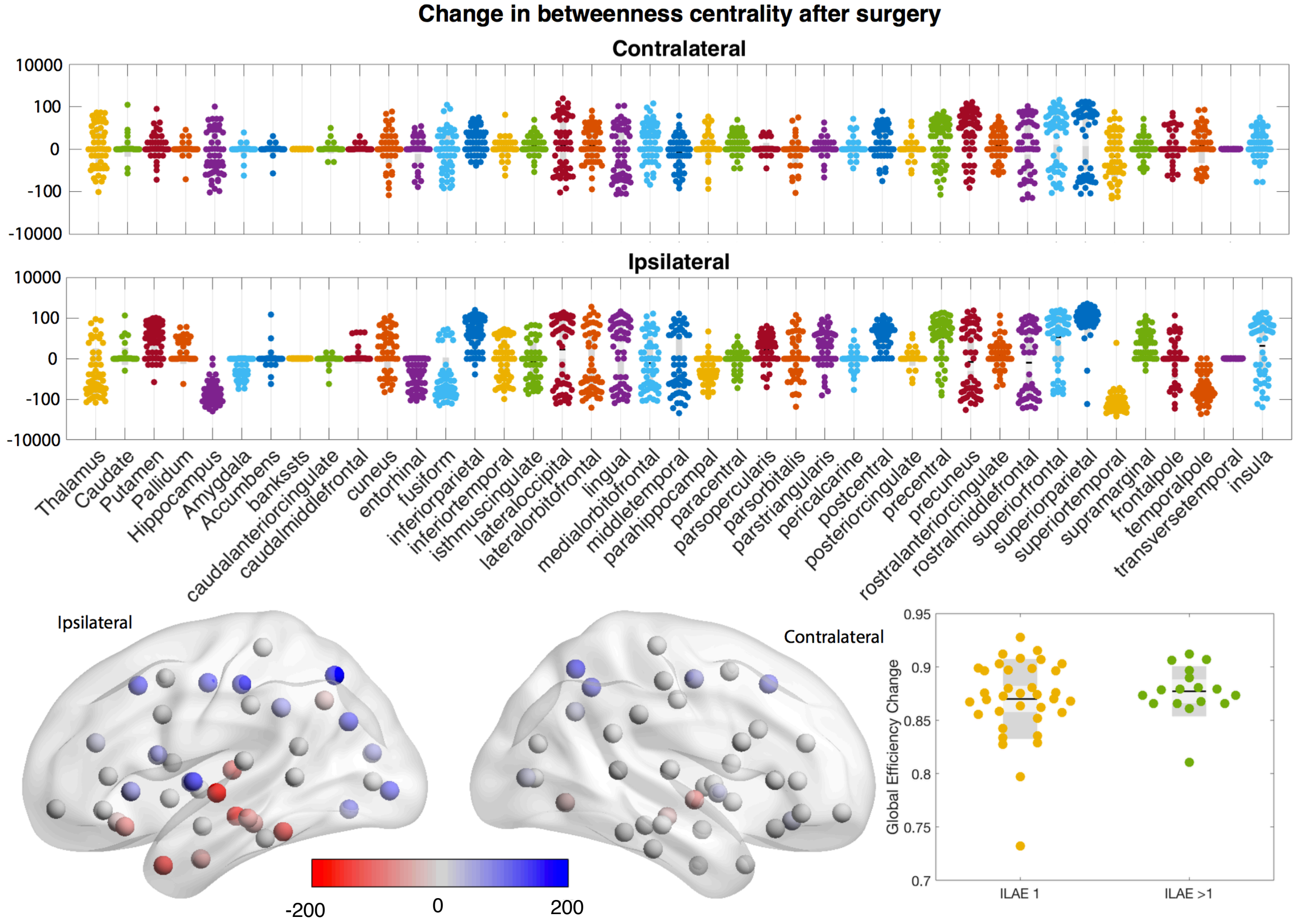

Supplementary Figure S6

Region clustering coefficient for both atlases.

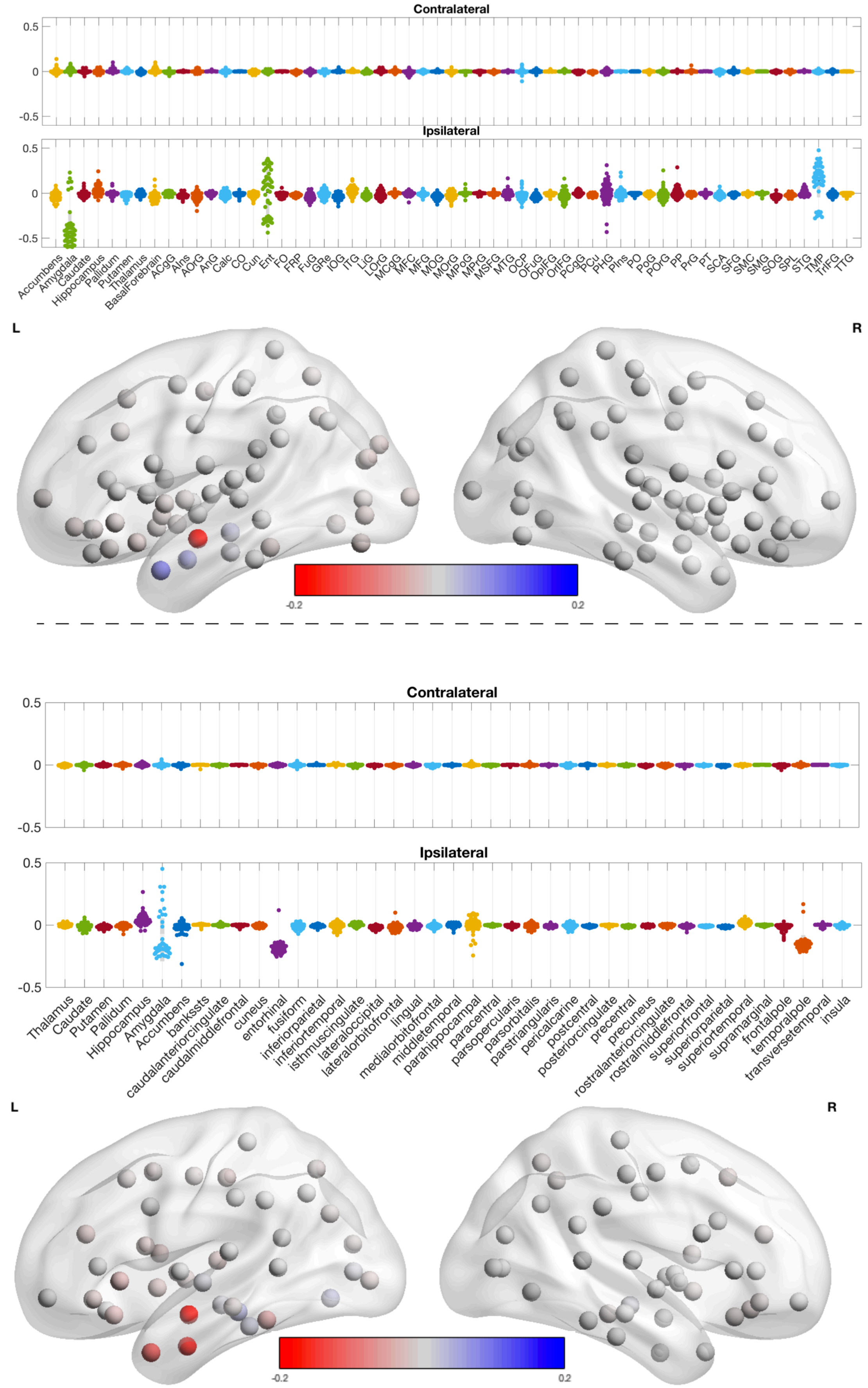

Supplementary Figure S7

Change in communicability. Most affected areas are ipsilateral amygdala, entorhinal cortex, and temporal pole (indicated by arrows) in both the GIF derived data (panel a), and the FreeSurfer derived data (b). None differed with respect to surgical outcome (p>0.05, Kruskal-Wallis test, FDR corrected; sigma=0.05).

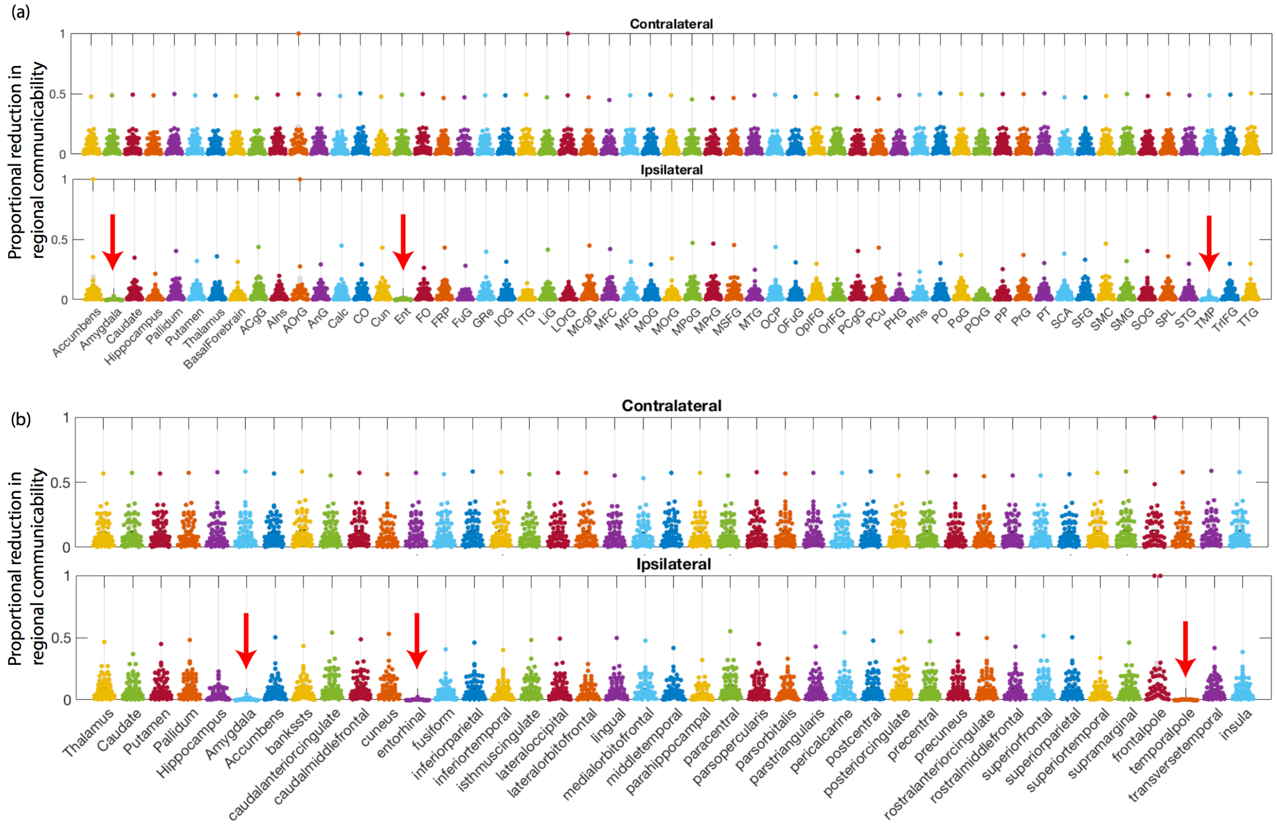

Supplementary Figure S8

Edge betweenness centrality for FreeSurfer derived atlas.

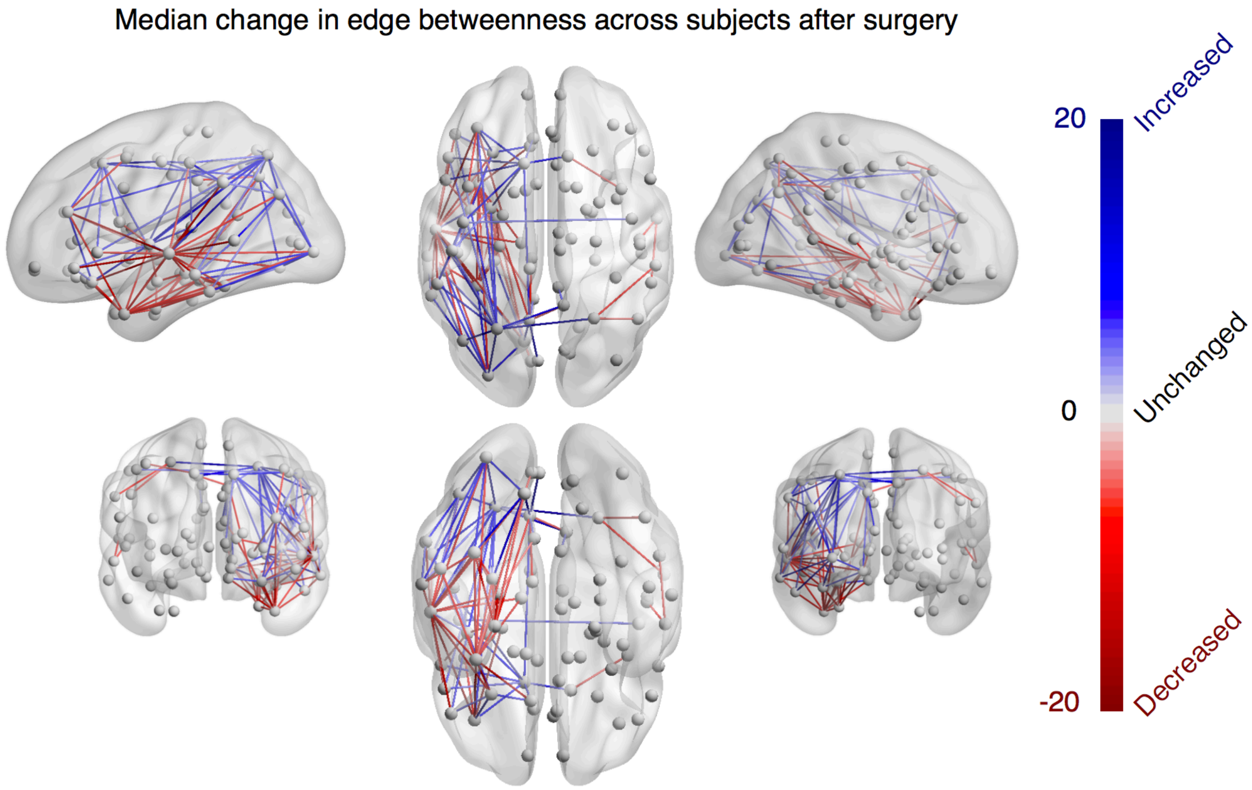

Supplementary Figure S9

Inter-rater agreement between manually drawn resection masks is high in both volume (a), dice similarity (b), and resulting node strength change (c).

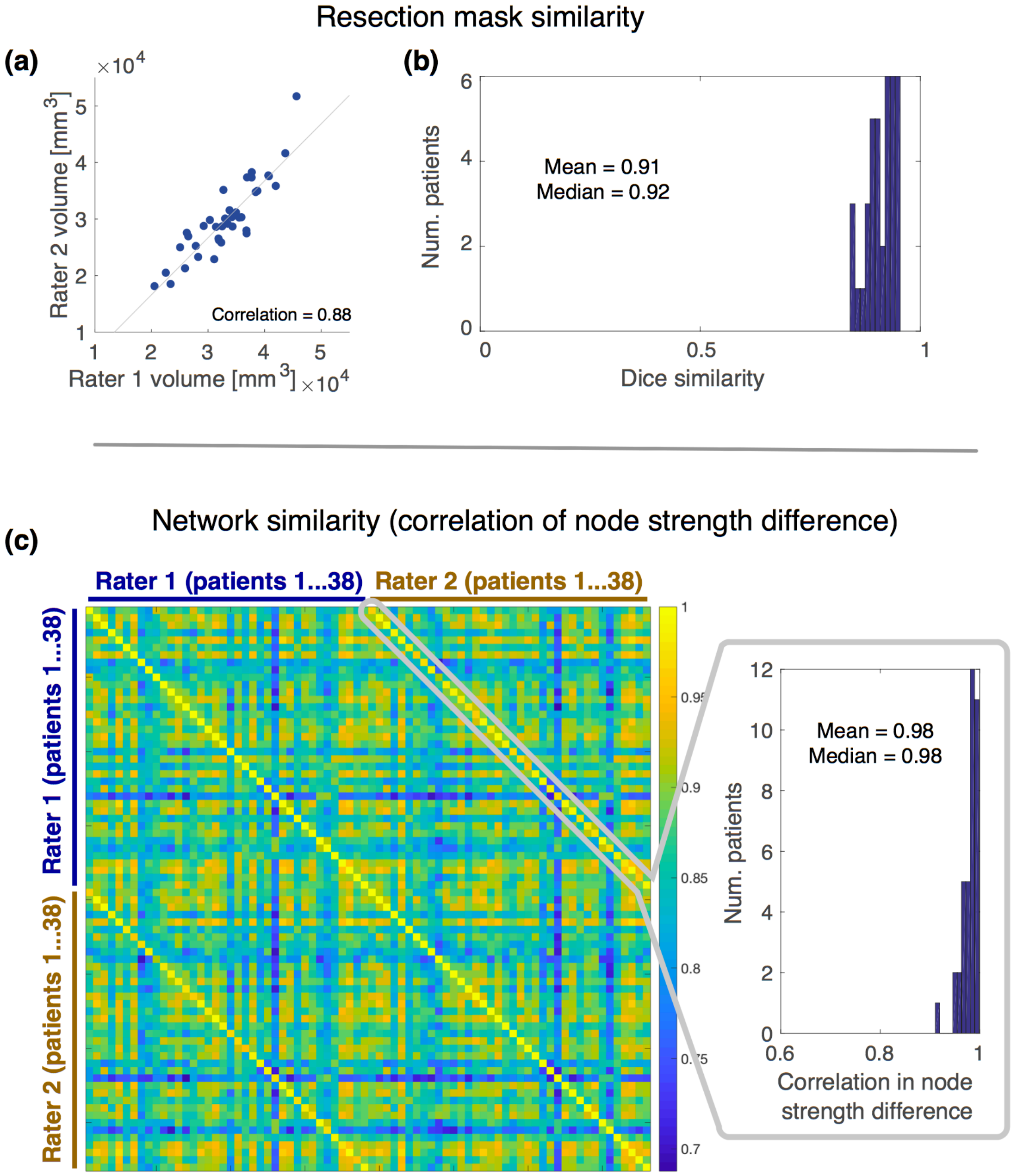

Supplementary results S10

1. Alternative atlas

The use of the alternative (FreeSurfer derived) network change measurements leads to an accuracy of 69.8% using 18 features.

Features selected from the freesurfer parcellation scheme.

- Ipsi-Caudate <-> ipsi-entorhinal
- Ipsi-Putamen <-> ipsi-entorhinal
- Ipsi-Putamen <-> ipsi-fusiform
- Ipsi-Amygdala <-> ipsi-fusiform
- Ipsi-Amygdala <-> ipsi-lateraloccipital
- ipsi-fusiform <-> ipsi-lateralorbitofrontal
- ipsi-fusiform <-> ipsi-middletemporal
- ipsi-fusiform <-> ipsi-precuneus
- ipsi-middletemporal <-> ipsi-rostralmiddlefrontal
- Ipsi-Hippocampus <-> ipsi-superiorparietal
- Ipsi-Amygdala <-> ipsi-superiorparietal
- ipsi-entorhinal <-> ipsi-superiorparietal
- Ipsi-Amygdala <-> ipsi-frontalpole
- Contra-Hippocampus <-> ipsi-temporalpole
- ipsi-inferiorparietal <-> ipsi-temporalpole
- Ipsi-Amygdala <-> ipsi-insula
- ipsi-cuneus <-> ipsi-insula
- ipsi-superiortemporal <-> contra-medialorbitofrontal

Confusion matrix using Freesurfer DK atlas.

		**Actual surgical outcome**	
		**Seizure free = 36**	**Not seizure free = 17**
**Predicted Outcome**	**Seizure free = 38**	True Positive=29	False Positive = 9
	**Not seizure free = 15**	False Negative = 7	True Negative = 8
	**Accuracy = 0.698**	Sensitivity = 0.856	Fall out = 0.529
		Miss Rate = 0.144	Specificity = 0.471

2. Data Normalisation

For completeness we here show our results when using GIF derived data upon normalisation (mean subtract and divide by standard deviation), and using preoperative data. All other methods used for the machine learning and classification are identical to those described in the main text.

Feature Scaling: change networks.

In the main manuscript we use a non-normalised version of the network change data since all values are bound in the same range (between 0 and 1). Normalisation of the feature vector through subtracting the mean of each feature and dividing by the standard deviation of each feature leads to an improved accuracy of 83%. However, this is at the cost of an increased number of features (33) and reduced specificity (52.9%) with a sensitivity of 97.2%.

Feature Scaling: preoperative networks.

Preoperative network values are unbound and span many orders of magnitude and are not bound between 0 and 1. Log_{10} streamline numbers are in the range of 0:6, whilst region volumes are in the range of 2,000 : 20,000. Normalisation of preoperative network features is therefore required. Here, we used normalised preoperative network features (connection strength, region strength, and region volume) as input to our machine learning algorithm. Doing this leads to a prediction accuracy of 69% (sensitivity:94%, specificity:17%) using 49 features. Note that in this scenario there are many more features used as input to the elastic net (2521).

Feature Scaling: preoperative & change networks combined

Finally, concatenating the normalised change network features with the normalised preoperative network features leads to a total of 3000 features as input to the elastic net algorithm. Feature selection here gives 72 features which yield an accuracy of 68% (sensitivity: 97.2%, specificity: 5.9%).

Supplementary Table S11

Extended patient data.

IDP	IsFemale	HasHS	AgeAtMRI	Outcome1	IsLTLE
'1001'	1	1	18.5	1	1
'1059'	1	1	19	1	1
'1120'	0	1	37.8	1	1
'1144'	1	1	27.4	1	1
'1152'	1	0	43.1	1	1
'1210'	0	0	52	1	1
'1252'	0	1	59	1	1
'1295'	0	1	56.4	1	1
'1347'	0	1	29.7	1	1
'0816'	0	1	42.8	1	1
'0844'	1	1	48.5	1	1
'0871'	1	1	45.6	1	1
'0878'	0	1	44.4	1	1
'0902'	1	1	30.8	1	1
'0909'	0	1	26.3	1	1
'0946'	0	1	31.9	1	1
'0977'	0	1	46.8	1	1
'0985'	0	0	33.7	1	1
'0986'	1	0	38.9	1	1
'0996'	0	1	20.8	1	1
'1065'	1	0	26.4	1	1
'1082'	0	0	40.6	1	1
'1012'	0	1	41.6	1	0
'1015'	1	0	19.5	1	0
'1022'	1	0	40.5	1	0
'1035'	1	1	38.2	1	0
'1041'	1	1	46.7	1	0
'1068'	1	1	46.8	1	0
'1083'	1	1	30.3	1	0
'1147'	1	1	47.3	1	0
'1148'	1	0	21.1	1	0
'1200'	1	0	23.6	1	0
'1236'	0	1	19.1	1	0
'0877'	1	1	41.6	1	0
'0968'	1	1	56.3	1	0
'1258'	0	0	40.3	1	0
'1102'	1	1	37.6	2	1
'1131'	0	1	38.9	2	1
'1151'	1	1	40.9	4	1
'1359'	1	1	32.6	2	1
'0873'	1	0	31.4	4	1
'0887'	1	1	47.6	5	1
'1179'	1	0	45	4	1
'1285'	0	1	35.9	3	1
'1005'	1	1	21.1	2	0
'1038'	1	0	42.3	2	0
'1052'	1	0	66.8	4	0
'1055'	0	0	44.9	4	0
'1118'	1	1	53.2	2	0
'1136'	1	0	45.3	4	0
'0983'	1	1	50.9	3	0
'0997'	1	0	27.3	2	0
'1260'	0	1	43.8	2	0

Supplementary Table S12a

Change in strength and significance between outcome groups (FS derived atlas)

Region Name	Mean change	S.D.of change	Median change	P value (KW test, unco	P value (permutation test, uncorrected)
'Ipsi-Thalamus'	0.76	0.10	0.78	0.62	0.83
'Ipsi-Caudate'	0.80	0.16	0.84	0.13	0.20
'Ipsi-Putamen'	0.89	0.07	0.91	0.55	0.44
'Ipsi-Pallidum'	0.93	0.09	0.95	0.57	0.62
'Ipsi-Hippocampus'	0.38	0.15	0.39	0.18	0.33
'Ipsi-Amygdala'	0.04	0.10	0.00	0.96	0.49
'Ipsi-Accumbens'	0.73	0.17	0.70	0.81	0.72
'Ipsi-bankssts'	1.00	0.00	1.00	0.80	0.35
'Ipsi-caudalanteriorcingulate'	0.98	0.04	1.00	0.81	0.67
'Ipsi-caudalmiddlefrontal'	1.00	0.02	1.00	0.58	0.62
'Ipsi-cuneus'	0.87	0.08	0.87	0.47	0.18
'Ipsi-entorhinal'	0.00	0.01	0.00	0.94	0.56
'Ipsi-fusiform'	0.57	0.12	0.58	0.92	0.80
'Ipsi-inferiorparietal'	0.95	0.08	0.97	0.11	0.42
'Ipsi-inferiortemporal'	0.70	0.16	0.73	0.82	0.48
'Ipsi-isthmuscingulate'	0.85	0.11	0.87	0.80	0.53
'Ipsi-lateraloccipital'	0.84	0.13	0.88	0.20	0.31
'Ipsi-lateralorbitofrontal'	0.66	0.15	0.67	0.66	0.43
'Ipsi-lingual'	0.78	0.08	0.79	0.78	0.88
'Ipsi-medialorbitofrontal'	0.83	0.08	0.84	0.78	0.77
'Ipsi-middletemporal'	0.77	0.16	0.82	0.40	0.08
'Ipsi-parahippocampal'	0.45	0.15	0.44	0.54	0.86
'Ipsi-paracentral'	0.99	0.02	1.00	0.29	0.92
'Ipsi-parsopercularis'	0.98	0.04	1.00	0.68	0.86
'Ipsi-parsorbitalis'	0.73	0.18	0.78	0.75	0.59
'Ipsi-parstriangularis'	0.91	0.11	0.96	0.21	0.87
'Ipsi-pericalcarine'	0.86	0.12	0.86	0.73	0.88
'Ipsi-postcentral'	0.99	0.02	0.99	0.17	0.70
'Ipsi-posteriorcingulate'	0.99	0.03	1.00	0.54	0.44
'Ipsi-precentral'	0.98	0.03	0.99	0.17	0.99
'Ipsi-precuneus'	0.91	0.04	0.90	0.80	0.35
'Ipsi-rostralanteriorcingulate'	0.95	0.06	0.96	0.89	0.88
'Ipsi-rostralmiddlefrontal'	0.84	0.07	0.86	0.38	0.38
'Ipsi-superiorfrontal'	0.96	0.03	0.97	0.23	0.97
'Ipsi-superiorparietal'	0.94	0.04	0.95	0.01	0.10
'Ipsi-superiortemporal'	0.54	0.12	0.53	0.42	0.86
'Ipsi-supramarginal'	1.00	0.01	1.00	0.39	0.12
'Ipsi-frontalpole'	0.85	0.14	0.84	0.26	0.88
'Ipsi-temporalpole'	0.00	0.01	0.00	0.97	0.43
'Ipsi-transversetemporal'	1.00	0.02	1.00	0.22	0.91
'Ipsi-insula'	0.82	0.10	0.82	0.33	0.19
'Contra-Thalamus'	0.94	0.06	0.95	0.08	0.04
'Contra-Caudate'	0.98	0.04	1.00	0.95	0.81
'Contra-Putamen'	0.97	0.04	0.99	0.45	0.82
'Contra-Pallidum'	0.97	0.05	1.00	0.83	0.25
'Contra-Hippocampus'	0.93	0.07	0.94	0.83	0.72
'Contra-Amygdala'	0.95	0.08	0.98	0.98	0.58
'Contra-Accumbens'	0.97	0.04	1.00	0.48	0.31
'Contra-bankssts'	1.00	0.01	1.00	0.49	1.00
'Contra-caudalanteriorcingulate'	0.99	0.02	1.00	0.05	0.07
'Contra-caudalmiddlefrontal'	1.00	0.00	1.00	1.00	0.79
'Contra-cuneus'	0.92	0.08	0.94	0.99	0.48
'Contra-entorhinal'	0.96	0.05	1.00	0.78	0.56
'Contra-fusiform'	0.97	0.03	0.98	0.69	0.45
'Contra-inferiorparietal'	0.99	0.01	1.00	0.56	0.88
'Contra-inferiortemporal'	1.00	0.01	1.00	0.77	0.37
'Contra-isthmuscingulate'	0.97	0.04	0.99	0.48	0.67
'Contra-lateraloccipital'	0.97	0.03	0.98	0.19	0.06
'Contra-lateralorbitofrontal'	0.98	0.03	1.00	0.23	0.19
'Contra-lingual'	0.94	0.05	0.94	0.49	0.47
'Contra-medialorbitofrontal'	0.96	0.04	0.97	0.94	0.80
'Contra-middletemporal'	0.99	0.02	1.00	0.33	0.33
'Contra-parahippocampal'	0.97	0.04	1.00	0.48	0.35
'Contra-paracentral'	0.99	0.02	1.00	0.55	0.75
'Contra-parsopercularis'	1.00	0.00	1.00	0.49	1.00
'Contra-parsorbitalis'	0.98	0.04	1.00	0.03	0.15
'Contra-parstriangularis'	0.99	0.02	1.00	0.95	0.41
'Contra-pericalcarine'	0.95	0.07	0.98	0.98	0.87
'Contra-postcentral'	0.99	0.02	1.00	0.09	0.89
'Contra-posteriorcingulate'	0.98	0.04	1.00	0.48	0.76
'Contra-precentral'	0.99	0.03	1.00	0.72	0.24
'Contra-precuneus'	0.96	0.03	0.97	0.57	0.37
'Contra-rostralanteriorcingulate'	0.97	0.03	0.98	0.09	0.27
'Contra-rostralmiddlefrontal'	0.97	0.03	0.98	0.42	0.72
'Contra-superiorfrontal'	0.98	0.02	0.99	0.62	0.51
'Contra-superiorparietal'	0.96	0.03	0.96	0.95	0.87
'Contra-superiortemporal'	0.98	0.03	0.99	0.79	0.84
'Contra-supramarginal'	1.00	0.00	1.00	1.00	1.00
'Contra-frontalpole'	0.96	0.06	0.99	0.14	0.66
'Contra-temporalpole'	0.97	0.04	0.98	0.91	0.92
'Contra-transversetemporal'	1.00	0.00	1.00	1.00	1.00
'Contra-insula'	0.99	0.02	1.00	0.26	0.24

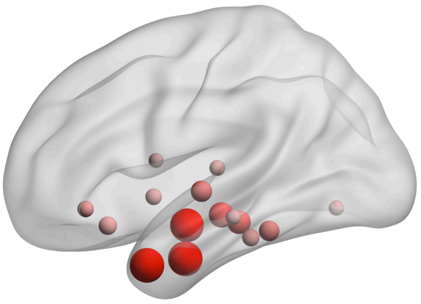

KW test is Kruskal-Wallis test.
Permutation test is 10,000 permutations for difference in mean values.

Groups were ILAE1 and ILAE>1.

Supplementary Table S12b

Change in strength and significance between outcome groups (GIF derived atlas)

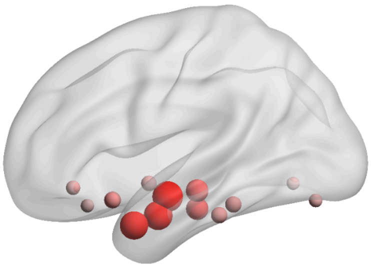

KW test is Kruskal-Wallis test.
Permutation test is 10,000 permutations for difference in mean values.

Groups were ILAE1 and ILAE>1.

Region Name	Mean change	S.D.of change	Median change	P value (KW test, uncorrected)	P value (permutation test, uncorrected)
'Ipsi-Accumbens'	0.77	0.14	0.76	0.86	0.91
'Ipsi-Amygdala'	0.04	0.08	0.00	0.42	0.76
'Ipsi-Caudate'	0.84	0.11	0.84	0.14	0.93
'Ipsi-Hippocampus'	0.38	0.15	0.36	0.06	0.51
'Ipsi-Pallidum'	0.95	0.07	0.99	0.87	0.31
'Ipsi-Putamen'	0.88	0.06	0.89	0.38	0.23
'Ipsi-Thalamus'	0.78	0.10	0.79	0.65	0.82
'Ipsi-BasalForebrain'	0.67	0.15	0.69	0.95	0.10
'Ipsi-ACgG'	0.96	0.05	0.96	0.69	0.19
'Ipsi-AIns'	0.88	0.08	0.90	0.59	0.37
'Ipsi-AOrG'	0.76	0.16	0.78	0.88	0.79
'Ipsi-AnG'	0.98	0.03	1.00	0.85	0.38
'Ipsi-Calc'	0.81	0.11	0.83	0.12	0.56
'Ipsi-CO'	0.98	0.05	1.00	0.21	0.92
'Ipsi-Cun'	0.86	0.07	0.87	0.12	0.61
'Ipsi-Ent'	0.09	0.09	0.07	0.26	0.80
'Ipsi-FO'	0.94	0.08	0.99	0.74	0.08
'Ipsi-FRP'	0.88	0.07	0.89	0.54	0.44
'Ipsi-FuG'	0.65	0.10	0.65	0.40	0.61
'Ipsi-GRe'	0.92	0.09	0.94	0.22	0.73
'Ipsi-IOG'	0.86	0.10	0.89	0.55	0.58
'Ipsi-ITG'	0.64	0.13	0.65	0.75	0.92
'Ipsi-LiG'	0.77	0.10	0.79	0.75	1.00
'Ipsi-LOrG'	0.69	0.17	0.74	0.57	0.67
'Ipsi-MCgG'	0.98	0.03	0.99	0.19	0.70
'Ipsi-MFC'	0.97	0.05	0.98	0.46	0.10
'Ipsi-MFG'	0.90	0.09	0.92	0.88	1.00
'Ipsi-MOG'	0.92	0.08	0.93	0.31	0.80
'Ipsi-MOrG'	0.75	0.13	0.79	0.39	0.14
'Ipsi-MPoG'	0.99	0.04	1.00	0.46	0.22
'Ipsi-MPrG'	0.99	0.02	1.00	0.36	0.86
'Ipsi-MSFG'	0.96	0.04	0.98	0.16	0.39
'Ipsi-MTG'	0.83	0.11	0.85	0.36	1.00
'Ipsi-OCP'	0.84	0.19	0.88	0.72	0.71
'Ipsi-OFuG'	0.78	0.12	0.77	0.88	0.17
'Ipsi-OpIFG'	0.99	0.03	1.00	0.54	0.84
'Ipsi-OrIFG'	0.78	0.17	0.85	0.04	0.38
'Ipsi-PCgG'	0.88	0.07	0.89	0.85	0.41
'Ipsi-PCu'	0.90	0.04	0.90	0.85	0.33
'Ipsi-PHG'	0.36	0.17	0.36	0.29	0.43
'Ipsi-PIns'	0.83	0.13	0.85	0.08	0.08
'Ipsi-PO'	0.99	0.02	1.00	0.43	0.80
'Ipsi-PoG'	0.98	0.03	0.99	0.07	0.22
'Ipsi-POrG'	0.60	0.17	0.63	0.66	0.70
'Ipsi-PP'	0.81	0.18	0.88	0.64	0.84
'Ipsi-PrG'	0.97	0.03	0.98	0.29	0.89
'Ipsi-PT'	0.99	0.03	1.00	0.29	0.22
'Ipsi-SCA'	0.83	0.09	0.85	0.39	0.40
'Ipsi-SFG'	0.96	0.03	0.96	0.95	0.99
'Ipsi-SMC'	0.98	0.03	0.99	0.51	0.74
'Ipsi-SMG'	1.00	0.01	1.00	0.99	0.42
'Ipsi-SOG'	0.87	0.09	0.91	0.18	0.25
'Ipsi-SPL'	0.95	0.04	0.97	0.09	0.13
'Ipsi-STG'	0.88	0.10	0.92	0.29	0.75
'Ipsi-TMP'	0.11	0.08	0.10	0.17	0.54
'Ipsi-TrIFG'	0.90	0.10	0.92	0.07	0.20
'Ipsi-TTG'	0.99	0.02	1.00	0.15	0.14
'Contra-Accumbens'	0.95	0.07	1.00	0.50	0.47
'Contra-Amygdala'	0.96	0.07	1.00	0.70	0.60
'Contra-Caudate'	0.98	0.03	1.00	0.42	0.56
'Contra-Hippocampus'	0.92	0.05	0.92	0.95	0.80
'Contra-Pallidum'	0.98	0.04	1.00	0.29	0.52
'Contra-Putamen'	0.98	0.03	0.99	0.88	0.76
'Contra-Thalamus'	0.95	0.06	0.96	0.58	0.12
'Contra-BasalForebrain'	0.94	0.08	0.97	0.70	0.89
'Contra-ACgG'	0.97	0.03	0.99	0.12	0.83
'Contra-AIns'	1.00	0.00	1.00	0.83	0.80
'Contra-AOrG'	0.98	0.04	1.00	0.72	0.49
'Contra-AnG'	1.00	0.00	1.00	0.60	0.65
'Contra-Calc'	0.94	0.08	0.96	0.40	0.99
'Contra-CO'	1.00	0.00	1.00	1.00	1.00
'Contra-Cun'	0.92	0.07	0.94	0.24	0.20
'Contra-Ent'	0.96	0.05	0.98	0.64	0.04
'Contra-FO'	1.00	0.00	1.00	1.00	0.04
'Contra-FRP'	0.98	0.02	0.98	0.28	0.50
'Contra-FuG'	0.97	0.03	0.98	0.58	0.89
'Contra-GRe'	0.99	0.03	1.00	0.87	0.06
'Contra-IOG'	0.99	0.02	1.00	0.13	0.92
'Contra-ITG'	0.99	0.02	1.00	0.53	0.26
'Contra-LiG'	0.95	0.04	0.95	0.73	0.26
'Contra-LOrG'	0.97	0.05	1.00	0.03	0.16
'Contra-MCgG'	0.98	0.03	1.00	0.36	0.35
'Contra-MFC'	0.98	0.03	1.00	0.48	1.00
'Contra-MFG'	0.99	0.02	1.00	0.07	0.79
'Contra-MOG'	0.99	0.01	1.00	0.31	0.57
'Contra-MOrG'	0.97	0.04	1.00	0.13	0.07
'Contra-MPoG'	1.00	0.01	1.00	0.71	0.17
'Contra-MPrG'	1.00	0.01	1.00	0.60	0.89
'Contra-MSFG'	0.99	0.02	1.00	0.63	0.46
'Contra-MTG'	1.00	0.01	1.00	0.69	0.59
'Contra-OCP'	0.97	0.05	1.00	0.72	0.34
'Contra-OFuG'	0.97	0.04	0.99	0.96	0.25
'Contra-OpIFG'	1.00	0.00	1.00	0.49	1.00
'Contra-OrIFG'	0.99	0.02	1.00	0.02	0.29
'Contra-PCgG'	0.97	0.04	0.99	0.72	0.38
'Contra-PCu'	0.95	0.04	0.96	0.18	0.18
'Contra-PHG'	0.97	0.04	0.98	0.48	0.33
'Contra-PIns'	1.00	0.01	1.00	0.41	0.89
'Contra-PO'	1.00	0.00	1.00	1.00	0.24
'Contra-PoG'	0.99	0.02	1.00	0.01	0.72
'Contra-POrG'	0.99	0.02	1.00	0.17	1.00
'Contra-PP'	1.00	0.02	1.00	0.92	0.48
'Contra-PrG'	0.99	0.03	1.00	0.46	0.01
'Contra-PT'	1.00	0.00	1.00	1.00	0.75
'Contra-SCA'	0.95	0.05	0.95	0.86	0.56
'Contra-SFG'	0.98	0.03	0.99	0.85	0.05
'Contra-SMC'	0.99	0.01	1.00	0.26	0.14
'Contra-SMG'	1.00	0.00	1.00	1.00	0.70
'Contra-SOG'	0.97	0.04	0.98	0.03	0.77
'Contra-SPL'	0.99	0.01	0.99	0.63	0.08
'Contra-STG'	1.00	0.01	1.00	0.11	0.24
'Contra-TMP'	0.96	0.03	0.97	0.98	0.06
'Contra-TrIFG'	0.99	0.02	1.00	0.44	1.00
'Contra-TTG'	1.00	0.00	1.00	1.00	0.60

Supplementary Table S13

Features selected when using the GIF derived parcellation scheme

- Ipsi Putamen <-> Ipsi Ent entorhinal area
- Ipsi Calc calcarine cortex <-> Ipsi ITG inferior temporal gyrus
- Ipsi FRP frontal pole <-> Ipsi LiG lingual gyrus
- Ipsi Amygdala <-> Ipsi MOG middle occipital gyrus
- Ipsi Cun cuneus <-> Ipsi MTG middle temporal gyrus
- Contra Cun cuneus <-> Ipsi PHG parahippocampal gyrus
- Ipsi Amygdala <-> Ipsi SCA subcallosal area
- Contra ACgG anterior cingulate gyrus <-> Ipsi TMP temporal pole
- Ipsi ACgG anterior cingulate gyrus <-> Ipsi TMP temporal pole
- Ipsi MSFG superior frontal gyrus medial segment <-> Ipsi TMP temporal pole
- Ipsi OrIFG orbital part of the inferior frontal gyrus <-> Ipsi TMP temporal pole
- Ipsi SMC supplementary motor cortex <-> Ipsi TMP temporal pole
- Contra SOG superior occipital gyrus <-> Ipsi TMP temporal pole
- Ipsi STG superior temporal gyrus <-> Ipsi TMP temporal pole
- Ipsi TMP temporal pole <-> Ipsi TrIFG triangular part of the inferior frontal gyrus

Supplementary Movie S14 (online only)

15 features from GIF derived atlas – three dimensional rendering of Figure 7 in the main manuscript.

Supplementary Results S15

Network synchronisability (Schindler et al, 2008, Khambhati et al, 2016) was affected by surgery in both the GIF, and freesurfer derived networks following surgery (left and right panels respectively). This did not significantly differ between outcome groups ($p>0.05$, Kruskal-Wallis test).

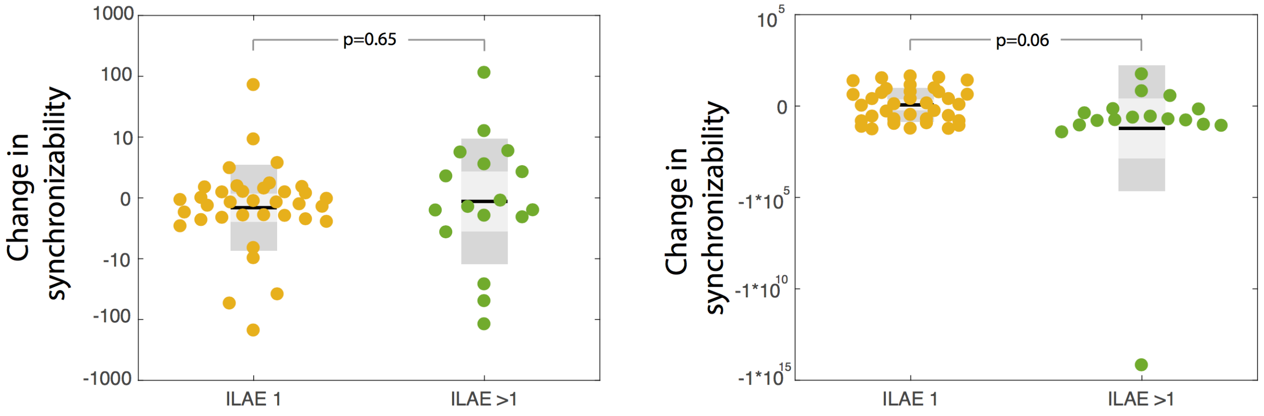

Khambhati, Ankit N., Kathryn A. Davis, Timothy H. Lucas, Brian Litt, and Danielle S. Bassett. "Virtual cortical resection reveals push-pull network control preceding seizure evolution." *Neuron* 91, no. 5 (2016): 1170-1182.

Schindler, Kaspar A., Stephan Bialonski, Marie-Therese Horstmann, Christian E. Elger, and Klaus Lehnertz. "Evolving functional network properties and synchronizability during human epileptic seizures." *Chaos: An Interdisciplinary Journal of Nonlinear Science* 18, no. 3 (2008): 033119.
